# Case Report: A Case of Moyamoya Syndrome Associated With Multiple Endocrine Neoplasia Type 2A

**DOI:** 10.3389/fendo.2021.703410

**Published:** 2021-11-11

**Authors:** Fumihiro Matano, Yasuo Murai, Atsushi Watanabe, Kazutaka Shirokane, Takehito Igarashi, Kazuo Shimizu, Takashi Shimada, Akio Morita

**Affiliations:** ^1^ Department of Neurosurgery, Nippon Medical School, Tokyo, Japan; ^2^ Division of Clinical Genetics, 3 Support Center for Genetic Medicine, Kanazawa University Hospital, Kanazawa, Japan; ^3^ Department of Endocrine Surgery, Nippon Medical School, Tokyo, Japan

**Keywords:** MEN2A, moyamoya syndrome, pheochromocytoma, *RET* gene, *RNF-213*

## Abstract

To the best of our knowledge, we report a case of MEN2A complicated by moyamoya syndrome. A 52-year-old woman presented with vertigo. Magnetic resonance angiography (MRA) revealed bilateral supraclinoid stenosis of the internal carotid artery and abnormal moyamoya-like vessels around the basal ganglia. She had a heterozygous variant of *RNF213*, which is the susceptibility gene for moyamoya disease. She had also previously received diagnoses of medullary thyroid carcinoma (MTC) at age 23 and left-sided pheochromocytoma (PHEO) at age 41. Genetic testing revealed heterozygosity for a mutation at codon 634 in exon 11 (TGC-TTC mutation; p.Cys634Phe) of the *Ret* gene. Intracranial vascular stenosis may have been caused by a genetic mutation of *RNF213* and hypersecretion of catecholamines by MEN2A. Physicians should recognize that MEN2A can be present with moyamoya syndrome.

## Introduction

Multiple endocrine neoplasia type 2A (MEN2A; OMIM #171400) is rare genetic disease that is characterized by medullary thyroid carcinoma (MTC), pheochromocytoma (PHEO), and multi-gland parathyroid tumors. If the condition remains uncontrolled, hormonal dysfunction, hypertensive crisis, cerebral infarction, hypercalcemia, and early death may occur (1).

Moyamoya disease is another rare genetic diseases; it is characterized by bilateral stenosis of the internal carotid artery. To prevent these complications, bypass surgery needed in some cases (2). On the other hand, moyamoya-like vascular changes can occur through autoimmune or endocrinology dysfunction or after radiation treatment; these changes represent secondary moyamoya disease, known as moyamoya syndrome or quasi-moyamoya disease.

In this report, we describe the first known case of MEN2A complicated by moyamoya syndrome and discuss the pathological processes of these conditions.

## Case Report

**Figure 1:**
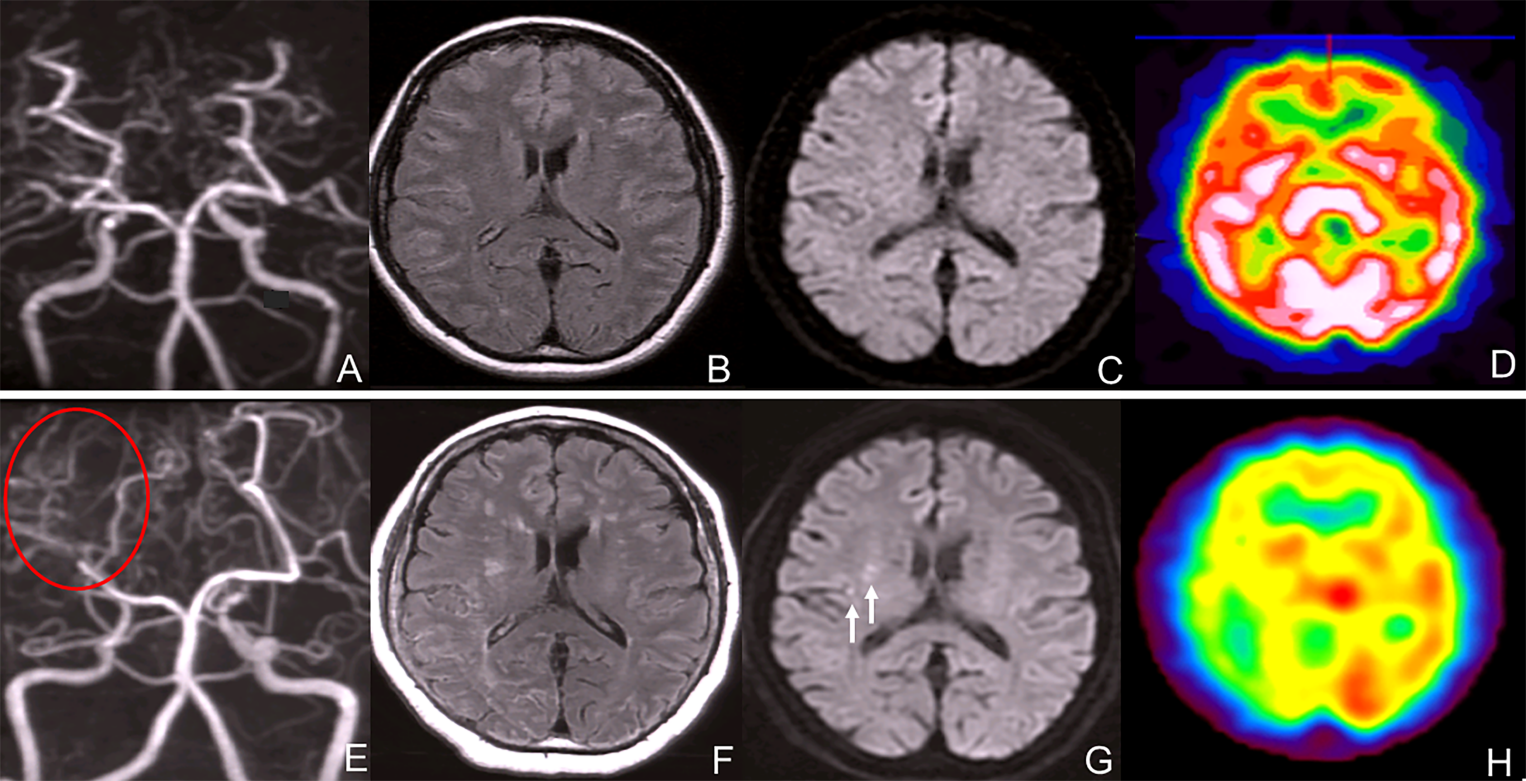
**(A)** Magnetic resonance angiography (MRA) revealed bilateral internal carotid artery stenosis. **(B)** MRI FLAIR showed no typical ischemic and hemorrhagic lesions in the brain. **(C)** MRI DWI showed no ischemic lesions. **(D)** SPECT showed no hypoperfusion or laterality of the blood flow. **(E)** Magnetic resonance angiography (MRA) revealed bilateral internal carotid artery stenosis and blood flow signal of PCA decreased. **(F)** MRI FLAIR showed bilateral ischemic lesions in the brain. **(G)** MRI DWI showed new ischemic lesions in the right deep white matter. (white arrows) **(H)** SPECT showed no hypoperfusion or laterality in the blood flow.

A 52 year-old woman visited our hospital complaining of vertigo without neurological deficit. She had a height of 157cm and a weigh of 61kg. She did not have an abnormal appearance of the face or tongue. She was taking levothyroxine sodium 300 µg/2x and simvastatin 10 mg/2x for hypothyroidism and dyslipidemia. Laboratory analysis revealed a carcinoembyronic antigen, level of 73.4 ng/mL (normal: <5.0 ng/mL); a calcitonin level of 1074 pg/mL (normal: 15-86 pg/mL); an adrenaline level of 107 pg/mL (normal <120 pg/ml); noradrenalin, 1154 pg/mL (normal: 60-500 pg/mL); and a dopamine level of 36 pg/mL (normal: <30pg/mL). All other blood cell counts and serum biochemistry parameters were normal. Magnetic resonance angiography (MRA) revealed bilateral supraclinoid stenosis of the internal carotid artery and abnormal moyamoya-like vessels around the basal ganglion (**Figure 1A**) but MRI FLAIR (**Figure 1B**) and DWI (**Figure 1C**) showed no specific ischemic or hemorrhagic lesions in the brain. SPECT showed no hypoperfusion or laterality (**Figure 1D**); using genomic DNA samples from the patients, we evaluated the polymorphism in c.14576G>A in the *RNF213*, a susceptibility gene for moyamoya disease, and the results showed a heterozygous variant of the *RNF213*.

**Figure 2:**
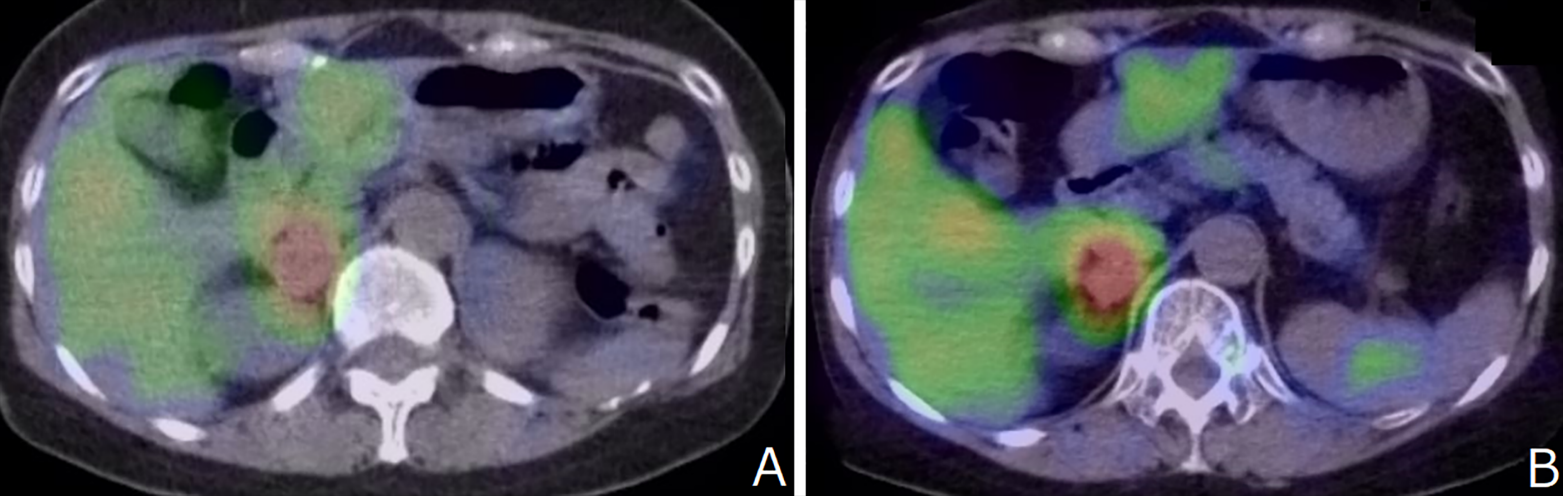
**(A)** No postoperative recurrence of the left adrenal lesion was observed, and the right adrenal lesion was unchanged and showed 123I-MIBG accumulation. **(B)** 123I-MIBG accumulation showed no change compared to that recorded 7 years ago.

The patient was diagnosed with stage T2N1M0 MTC at another hospital at age 23; the condition was managed surgically with total thyroidectomy and left central neck D2a dissection. At age 41, she was diagnosed with left-sided pheochromocytoma (PHEO) at our hospital, for which she underwent surgery. No postoperative recurrence of the left adrenal lesion was observed, and the right adrenal lesion was unchanged and showed 123I-MIBG accumulation. (**Figure 2A**)

**Figure 3:**
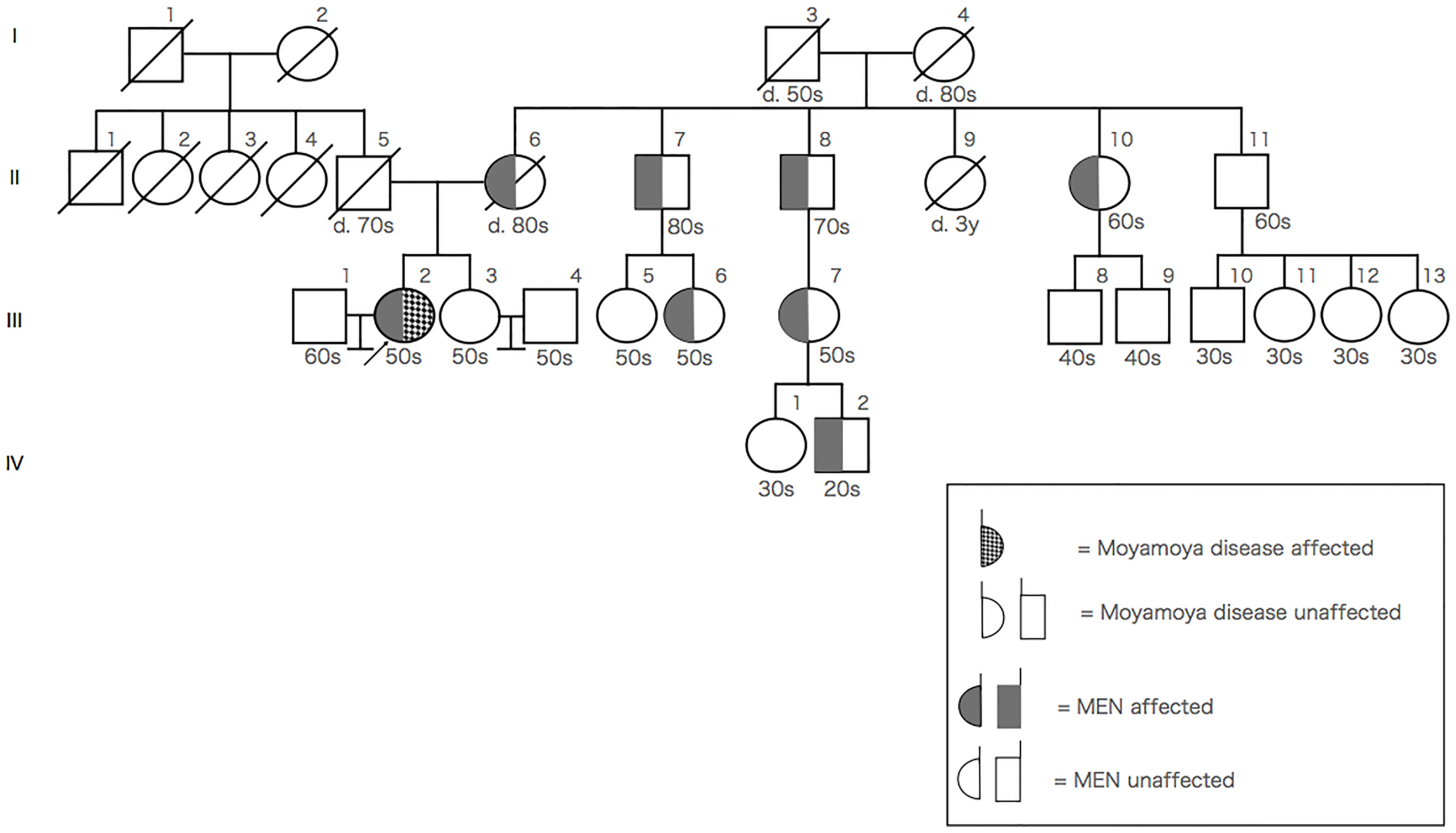
Pedigree chart of the patient’s family members (arrow indicates the index patient).

Genetic testing, performed after she provided informed consent, revealed heterozygosity for a mutation at codon 634 in exon 11 (TGC-TTC mutation; p.Cys634Phe) of the *RET gene*. The patient’s mother underwent thyroid surgery and bilateral pheochromocytomas were observed.　MEN2A patients are on the maternal side, but not on the paternal side. Moyamoya disease and stroke patients are neither on the paternal side nor the maternal side. The pedigree chart is shown in **Figure 3**. There was a possibility that certain family members of the patient could not develop the moyamoya disease; however, this could not be confirmed because screening tests, such as MRA, were unavailable. On the basis of genetic analyses, imaging findings, and a history of clonal presence of multiple endocrine neoplasia type 2A (MEN2A; OMIM # 171400), MEN2A with moyamoya syndrome was diagnosed.

After 7 years, MRA revealed bilateral supraclinoid stenosis of the internal carotid artery as observed previously; however, right PCA blood flow decreased. (**Figure 1E**) MRI FLAIR showed an increase in ischemic lesions in the bilateral hemiparesis. (**Figure 1F**) MRI DWI showed new ischemic lesions in the right hemiparesis. (**Figure 1G**) SPECT showed progressive hypoperfusion in the right hemiparesis (**Figure 1H**). Laboratory analysis revealed a carcinoembyronic antigen, level of 105.8 ng/mL (normal: <5.0 ng/mL); a calcitonin level of 440.7 pg/mL (normal: 15-86 pg/mL); an adrenaline level of 210 pg/mL (normal <120 pg/ml); noradrenalin, 1010 pg/mL (normal: 60-500 pg/mL); and a dopamine level of 18 pg/mL (normal: <30 pg/mL). All other blood cell counts and serum biochemistry parameters were normal. 123I-MIBG accumulation showed no changing compared to 7 years ago. (**Figure 2B**) We planned STA-MCA bypass; however, it is pending because of the Corona virus disease 19 pandemic.

## Discussion

Our patient represents the first known case of MEN2A complicated by moyamoya syndrome. Physicians should recognize that the hormonal dysfunction sometimes occurs with moyamoya syndrome.

MEN2A is caused by germline missense mutations in the proto-oncogene rearranged during transfection (*RET*) proto-oncogene, localized to chromosome 10, arising most commonly (in 80%-90% of cases) from mutations in codon 634 of *RET*. Moyamoya disease is characterized by bilateral supraclinoid internal carotid artery stenosis and an abnormal vascular network around the basal ganglion. The pathogenesis of moyamoya disease is unknown. The disease is observed predominantly among the East Asian population (3), and the prevalence of moyamoya disease appears to be slightly lower among Chinese, compared with Koreans or Japanese (4). Recent studies have increasingly reported this disease in adults and in the Western population (5). Most of the cases of moyamoya disease are sporadic, but there is a small percentage that is familial; ring finger protein 213 (*RNF213*), encoded by chromosome 17, has been implicated as a cause for the disease (6).

PHEO is associated with cerebral infarction and vasospasm, which is secondary to excessive catecholamine secretion. The catecholamine pathway is a primary regulator of cerebrovascular tone and cerebral blood flow, and the majority of its effects are mediated though alpha-adrenergic receptors. These receptors are abundant in the cerebral arteries and are stimulated by catecholamines, which leads to increased intracellular calcium concentration and vascular contraction. Some reports have concluded that cerebral infarction caused by PHEO is vasospasm that results from excessive catecholamine secretion (7).

The *RNF213* gene has been identified as a susceptibility gene for moyamoya disease. The polymorphism, p.R4810K, is found not only in 80%- 90% of Japanese patients, but also found in 1%-2% of healthy Japanese patients. In other words, most carriers of the polymorphism do not develop moyamoya disease, and moyamoya disease is considered to be a multifactorial disease in which not only the gene but also some secondary factors, such as inflammation, are involved in the development of the disease (8).

Adult onset cases of moyamoya disease tend to be hemorrhagic and occur between the ages of 30 and 45 (9). In our case, the onset was ischemic and relatively late in terms of age. We mainly suspected that cerebrovascular occlusion complicated with moyamoya-like vessels (i.e., moyamoya syndrome) in our patient was caused by excessive catecholamine secretion by the PHEO. This is because our case was an atypical case of moyamoya disease, and it is quite possible that the ischemic lesion progressed gradually due to catecholamine-stimulated MEN2A, despite there originally being an element of *RNF213* mutation. In our patient, conversely, in moyamoya disease, although a number of genetic abnormalities have been identified, no studies have demonstrated an association between moyamoya disease and *RET*, and the moyamoya-like vessels in our patient were real moyamoya disease caused by *RNF213* mutation, which just coincidentally complicated MEN2A.

We could not identify the real cause of moyamoya syndrome in this case, but both pathological processes are extremely rare. To prevent progressive arterial stenosis in moyamoya syndrome, the most important treatment is monitoring and controlling the disease as if it were an autoimmune disorder or endocrine disease. In the past, some cases of arterial stenosis have improved after medical treatment; therefore, the first strategy is medical treatment (10). If progressive vascular occlusion or bleeding had occurred in our patient, we would have considered surgical therapy, such as bypass.

## Conclusions

As this is the first report of this case, future studies and reports are warranted to establish a complete association. However, the concomitant hypersecretion of catecholamines in a case with genetic susceptibility to *RNF213* may have resulted in a clinical phenotype of intracranial vascular stenosis. Physicians should recognize that MEN2A can be complicated with moyamoya syndrome.

## Ethics Statement

Ethical review and approval was not required for the study on human participants in accordance with the local legislation and institutional requirements. The patients/participants provided their written informed consent to participate in this study. Written informed consent was obtained from the individual(s) for the publication of any potentially identifiable images or data included in this article.

## Author Contributions

FM: Manuscript review. AW: Data analysis. KShir: Manuscript review. TI: Data analysis. KShim: Manuscript review. TS: Manuscript review. AM: Manuscript review. All authors contributed to the article and approved the submitted version.

## Funding

This work was supported by Acknowledgments Formatted: Superscript10 1 This work was supported by JSPS KAKENHI Grant-in-Aid for Scientific Research (C) Number 182 K(K)09008.

## Conflict of Interest

The authors declare that the research was conducted in the absence of any commercial or financial relationships that could be construed as a potential conflict of interest.

## Publisher’s Note

All claims expressed in this article are solely those of the authors and do not necessarily represent those of their affiliated organizations, or those of the publisher, the editors and the reviewers. Any product that may be evaluated in this article, or claim that may be made by its manufacturer, is not guaranteed or endorsed by the publisher.

## Abbreviations

MEN2A, Multiple endocrine neoplasia type 2A; MMD, Moyamoya disease; MRA, Magnetic resonance angiography; MRI, Magnetic resonance imaging; MTC, Medullary thyroid carcinoma; PHEO, Pheochromocytoma.
